# 

**Published:** 1852-03

**Authors:** 


*University of the City of New York.*—It is stated that Dr. Mott is to resume his connection with this Institution, Dr. Post remaining and giving a portion of the course on Surgery. It is also understood that Dr. Van Buren, of New York, is to take the chair of Anatomy made vacant by the death of Dr. Patterson.

---

*New Work on Surgery. Importing Book Store, etc., of H. Balliere.*—We would invite the attention of our readers to the new work on Surgery, to be issued in parts, by H. Balliere, of New York. Persons desirous of importing foreign works, instruments, preparations, etc., can do so with every regard to cheapness and dispatch, through the agency of Mons. Balliere. See advertisement on second page cover.

---

*To Readers and Publishers.*—Several valuable works have been received which we have not, as yet, been able to examine, but which will receive early attention.

---

*Dr. John Bell.*—Dr. Bell has resigned the chair of practice in the Cincinnati Medical College, and returns to Philadelphia.

